# Vegetation-fire feedback reduces projected area burned under climate change

**DOI:** 10.1038/s41598-019-39284-1

**Published:** 2019-02-26

**Authors:** Matthew D. Hurteau, Shuang Liang, A. LeRoy Westerling, Christine Wiedinmyer

**Affiliations:** 1 Department of Biology, University of New Mexico, Albuquerque, USA; 2 Department of Natural Resources and Environmental Sciences, University of Illinois, Urbana, USA; 3 School of Engineering, University of California, Merced, USA; 4 Cooperative Institute for Research in Environmental Sciences, University of Colorado, Boulder, USA

## Abstract

Climate influences vegetation directly and through climate-mediated disturbance processes, such as wildfire. Temperature and area burned are positively associated, conditional on availability of vegetation to burn. Fire is a self-limiting process that is influenced by productivity. Yet, many fire projections assume sufficient vegetation to support fire, with substantial implications for carbon (C) dynamics and emissions. We simulated forest dynamics under projected climate and wildfire for the Sierra Nevada, accounting for climate effects on fuel flammability (static) and climate and prior fire effects on fuel availability and flammability (dynamic). We show that compared to climate effects on flammability alone, accounting for the interaction of prior fires and climate on fuel availability and flammability moderates the projected increase in area burned by 14.3%. This reduces predicted increases in area-weighted median cumulative emissions by 38.3 Tg carbon dioxide (CO_2_) and 0.6 Tg particulate matter (PM1), or 12.9% and 11.5%, respectively. Our results demonstrate that after correcting for potential over-estimates of the effects of climate-driven increases in area burned, California is likely to continue facing significant wildfire and air quality challenges with on-going climate change.

## Introduction

Continued warming is projected to substantially increase the area burned by wildfire in regions that currently burn^1,2^. However, these approaches assume stationarity in the climate-fire relationship^3^, but see Turco *et al*.^4^. While regionally, climate controls vegetation growth and fire synchrony^5^, at local scales vegetation and topography influence patch flammability^6^. Thus, the combined effects of forest response and area burned to future climate are a significant source of uncertainty for projecting carbon dynamics and fire emissions^7,8^.

As temperature has increased in the western US, forested area burned has increased by more than 1200% over the past four decades^9^. From 2001–2008, mean annual carbon emissions from western US forest fires were 17.9 Tg yr^−1^ ^10^. Ongoing climate change is projected to increase area burned because higher temperatures lead to increased fuel aridity, making vegetation more available to burn^2,10^. This has the potential to substantially increase emissions of carbon and other criteria air pollutants, with significant health implications^7,11,12^. Yet, fire is a self-limiting process and the influence of changing climate on post-fire vegetation recovery is likely to alter the way subsequent fires interact with these ecosystems^6,13,14^.

Changing climate alone can alter forest composition and productivity through the combined effects of higher temperature and drought^15^. Under projected climate, fire can act as a catalyst for forest change because climatic tolerance differs among species and can vary as a function of age for trees, with juveniles typically having a narrower climate niche^16^. Thus, the effects of the climate-fire interaction on vegetation are likely to create feedbacks to subsequent fire size and emissions.

The forests of the Sierra Nevada of California were historically shaped by fire, with low- and mid-elevation forests having fire return intervals on the order of years to decades and high-elevation forests on the order of decades to centuries^17^. When a forest burns at higher frequency, fires tend to be less severe and the resultant emissions are lower^10^. Further, fire-induced tree mortality is lower and the amount of carbon taken up and stored by ecosystems for a sustained period is higher^18^. However, many of the fire-prone forests in the Sierra are in a higher biomass state as a result of fire-exclusion, and this has increased the proportion of the landscape that is impacted by more severe fire^19^. When a forested area experiences high-severity fire, it has an increased probability of a subsequent fire being high-severity^20^; which has implications for both future carbon uptake and emissions if the post-fire community that develops has a lower biomass density^21^.

Prior research in the Sierra Nevada has demonstrated that climate-wildfire interactions have the potential to fundamentally alter the distribution of tree species, the extent of forest cover, and carbon dynamics^16,21^. To improve the linkage between prior and future fire events as moderated by vegetation and quantify the influence of this feedback on carbon dynamics and wildfire emissions in the Sierra Nevada, we used a process-based forest landscape model (LANDIS-II) to simulate forest and fire dynamics for three transects across the Sierra Nevada Mountains under projected climate (see Methods, Supplementary Fig. S1). We conducted simulations that assumed climate only affected area burned distributions via fuel flammability (static) and area burned distributions that accounted for the interaction of climate and prior fire events on both fuel flammability and availability (dynamic) using climate projections from three climate models forced with the medium-high, A2 emission scenario. The dynamic simulations used biomass layers that were impacted by fires simulated in the prior decade to re-estimate fire size distributions for the subsequent decade. Prior fire events impacted both the distribution of biomass and the fuel model that contributes to the determination of fire spread characteristics.

**Figure 1:**
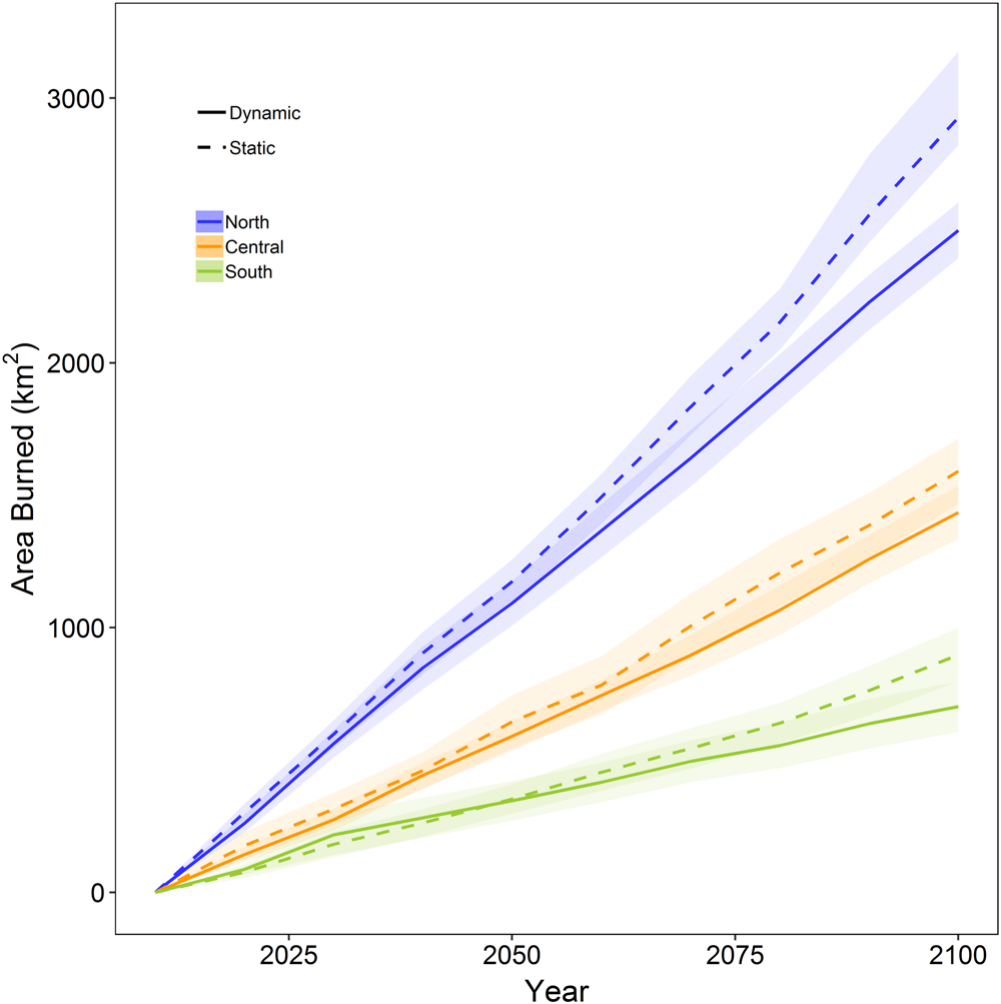
Cumulative area burned for a latitudinal gradient of transects in the Sierra Nevada under projected climate. The dynamic simulations include decadal re-estimated area burned distributions that account for prior fire events and projected climate. The static simulations include area burned distributions estimated only on projected climate. The lines are means and the shading 95^th^ percentile confidence intervals from the 10 replicate simulations for each of the three climate scenarios.

Accounting for the bottom-up control on area burned exerted by the effect of prior wildfire on vegetation available to burn, we found reductions in cumulative area burned ranging from −9.8 to −21.8% across the three transects (Fig. 1). Scaled to the mountain range, this equates to a reduction in area-weighted mean cumulative area burned of 2563.7 km^2^ through 2100, a −14.3% change in cumulative area burned when accounting for the effects of prior wildfires on fire size.

The cumulative area burned results were driven by the effect of prior burns on limiting the size of individual fire events because we held all other parameters constant between the two scenarios. The north transect showed a larger decrease in area burned under the dynamic scenario than the central and south transects because the north transect is less topographically diverse and a larger fraction of the land area is in lower elevation vegetation types that are available to burn over a larger proportion of the year. There was no statistical difference between fire size distributions during the first three decades of the simulation period, with means of 1060 ha for dynamic and 1105 ha for static simulations (Supplementary Fig. S2). However, by the final three decades of the simulation period (2070–2099), accounting for the influence of prior fires on subsequent fire size yielded a 20.9% reduction in mean fire size for the dynamic simulations (Supplementary Table S1).

**Figure 2:**
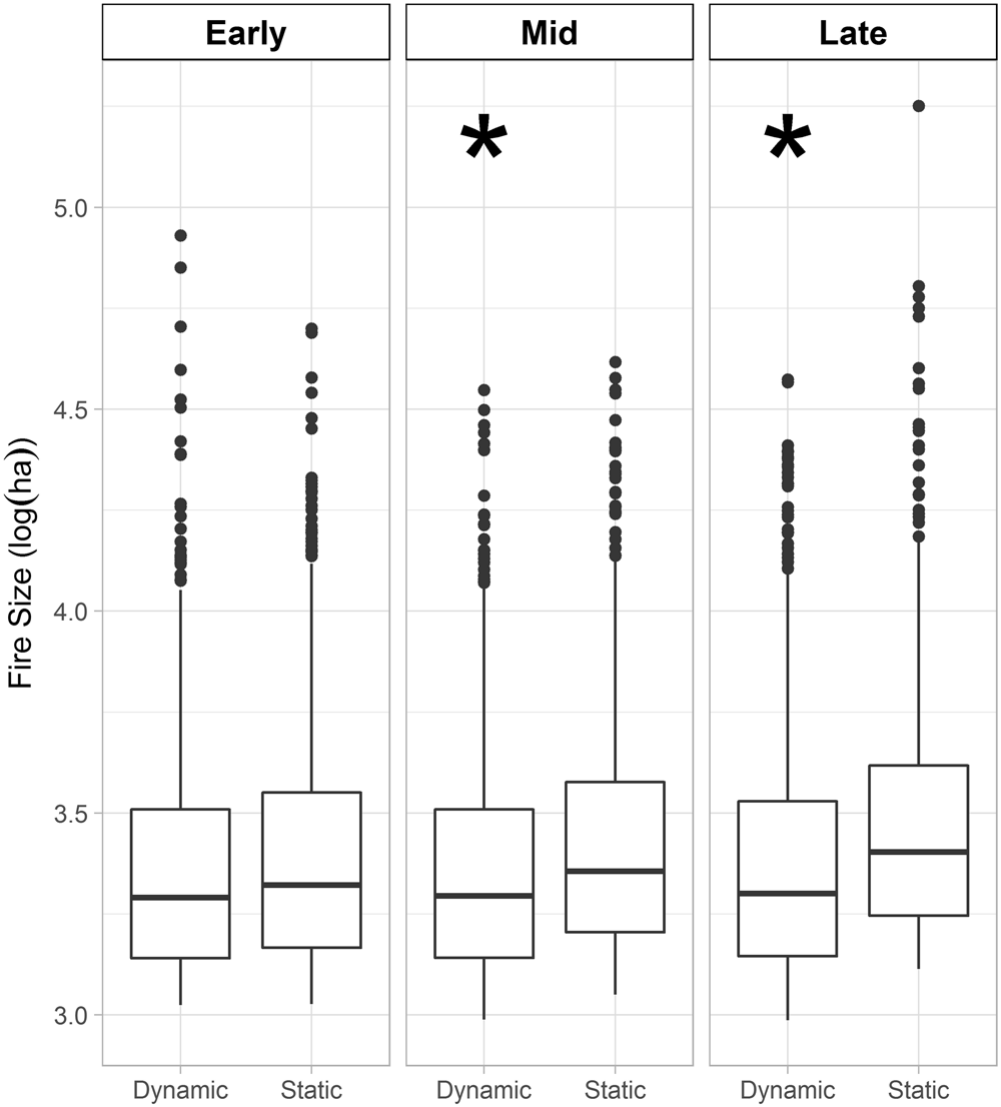
Upper quartile of fire size distributions by time period. The periods are 2010–39 (early), 2040–69 (mid), and 2070–99 (late). An asterisk denotes that the static distribution is significantly greater (*p < 0.001) than the dynamic distribution for that time period. The dynamic simulations include decadal re-estimated area burned distributions that account for prior fire events and projected climate. The static simulations include area burned distributions estimated only on projected climate. Fire sizes are from 10 replicate simulations for each of the three climate scenarios. Boxes represent the median and quartiles, whiskers the non-outlier range, and dots the outliers.

**Figure 3:**
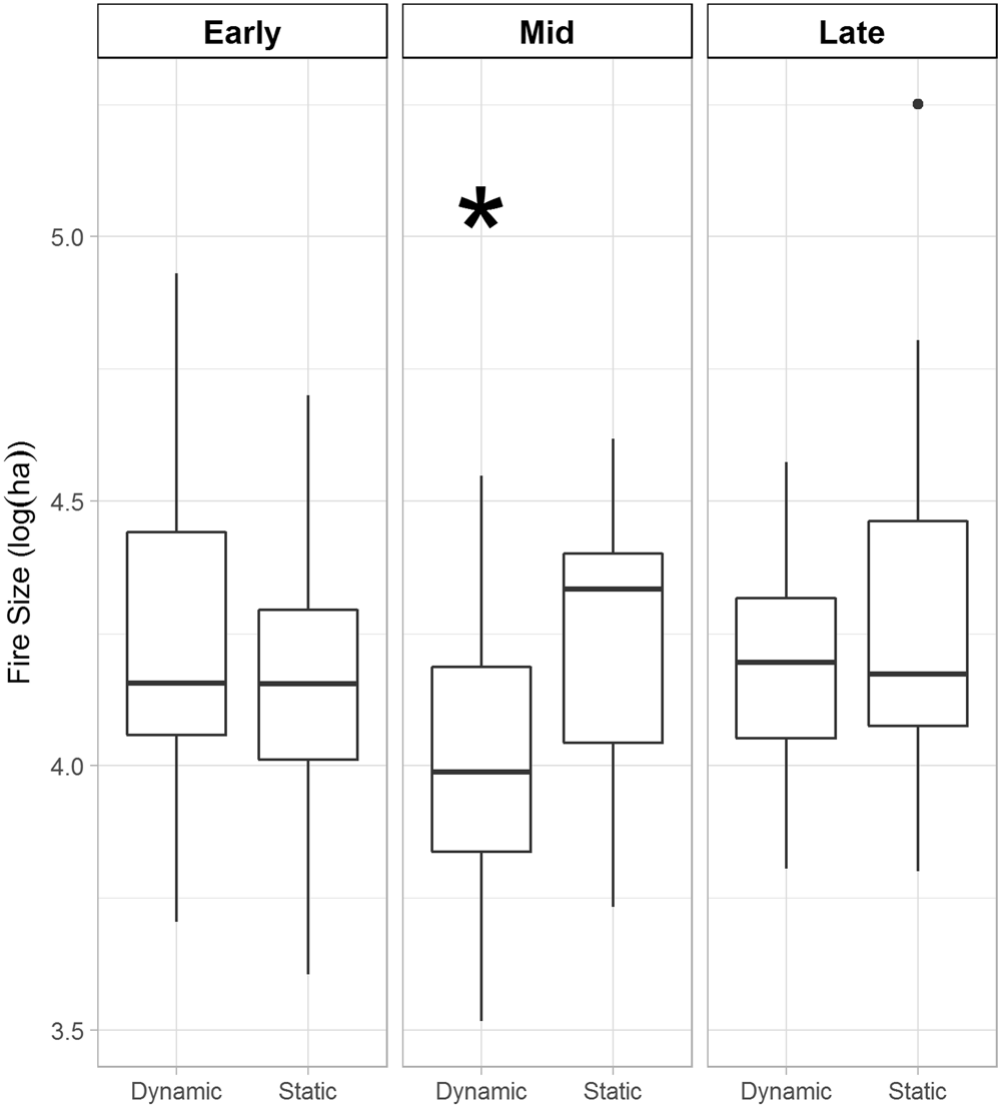
Maximum fire size distributions by time period. The periods are 2010–39 (early), 2040–69 (mid), and 2070–99 (late). An asterisk denotes that the static distribution is significantly greater (*p < 0.01) than the dynamic distribution for that time period. The dynamic simulations include decadal re-estimated area burned distributions that account for prior fire events and projected climate. The static simulations include area burned distributions estimated only on projected climate. Fire sizes are from 10 replicate simulations for each of the three climate scenarios. Boxes represent the median and quartiles, whiskers the non-outlier range, and dots the outliers.

Accounting for the vegetation-fire feedback had the largest effect on the upper quartile of fire size, with this distribution being significantly lower under the dynamic scenario during mid- and late-century periods (Fig. 2). The distributions of the largest wildfires, which are often the most costly in terms of fire suppression efforts, were only significantly different during the mid-century period (Fig. 3, Supplementary Table S2). During the early-century period, the mean percentage of area burned was similar for the dynamic (15.2%) and static (15.7%) simulations. Accounting for the influence of prior area burned demonstrates the constraints imposed on the size of upper quartile and largest wildfires are substantial.

Emissions under the dynamic scenario, when area-weighted for the entire mountain range, were substantially lower than the static scenario (Table 1). Nonetheless, median cumulative CO_2_ and methane (CH_4_) emissions for the dynamic scenario were 257.7 Tg and 0.9 Tg, respectively. The 2013 Rim fire, which burned more than 1040 km^2^ in the central Sierra Nevada, released approximately 12 Tg CO_2_e^22^. The median cumulative CO_2_ and CH_4_ emissions from our dynamic simulations are equivalent to one Rim fire occurring on average every 3.8 years through late-century. Even when accounting for the prior area burned feedback in estimating future fire size, these results suggest that California will continue to be challenged by increasing emissions of greenhouse gases and criteria air pollutants with climate change.

**Table 1:** Interquartile ranges of area-weighted cumulative wildfire emissions of carbon dioxide (CO_2_), carbon monoxide (CO), methane (CH_4_), submicron aerosols (PM1), and organic aerosols (OA) for the entire mountain range.

Constituents	Dynamic (Tg)	Static (Tg)
CO_2_	221–277	264–328
CO	13.6–17.0	16.2–20.0
CH_4_	0.7–0.9	0.9–1.1
PM1	4.0–4.9	4.7–5.8
OA	3.6–4.6	4.4–5.5

The dynamic simulations include decadal re-estimated area burned distributions that account for prior fire events and projected climate. The static simulations include area burned distributions estimated only on projected climate.

While our results demonstrate that accounting for the vegetation-fire feedback leads to a lower rate of increase in area burned with changing climate, area burned remains likely to increase. Our simulations estimate that the vegetation-fire feedback is only responsible for 7.5% of the cumulative area burned because vegetation re-growth happens with sufficient speed that the fuel limitation effects from fire are short-lived^13^. While accounting for this feedback does help constrain future wildfire projections, it does not account for the severity of fire that occurs or the effect a fire has on the ecosystem.

Fire alters forest C stocks and rates of uptake, with C stock loss and the length of time a forest is a C source increasing with increasing fire severity^23^. The severity with which a forest fire burns is a function of fuels (forest structure and biomass density), topography, and weather^6^. Given that we held the fire weather distribution constant between the scenarios, the only potential influence on fire severity was the effects of prior fire events on fuels. As a result, we found little difference in mean fire severity between the two scenarios (Supplementary Figs S3–S4) Thus, area burned was the sole factor influencing C storage on the landscape. Total ecosystem carbon (TEC) did not begin to deviate between the two scenarios until mid-century (Supplementary Fig. S5). By 2100, the dynamic scenario mean TEC was 729 Tg (±7.9, 95% CI) and the static scenario mean was 717 Tg (±10.6, 95% CI). This is largely driven by the fact that even with the vegetation-fire feedback, 44% of the mountain range burns over the simulation period.

In addition to the emissions challenges on-going climate change poses to California, increasing area burned, especially if the fraction of high-severity fire increases, presents additional challenges for meeting the State’s climate mitigation target. However, this is not a foregone conclusion. Prior research has shown that managing the low- and mid-elevation forests in the Sierra Nevada with frequent burning can significantly reduce the area burned by high-severity wildfire and wildfire emissions^18^. The rate at which these treatments are applied can significantly alter the amount of C emitted to the atmosphere as area burned by wildfire increases through this century. Given we found no difference in area burned through 2039 for our two scenarios and the potential for regular burning to alter the way wildfire interacts with forests, early action to reduce the chance of high-severity fire could yield significant gains in reducing emissions.

Our results demonstrate the importance of adequately characterizing the relationship between climate, ecosystems, and area burned over large landscapes for understanding how additional climate warming will alter future wildfire and carbon. Future wildfire and carbon trajectories in the Sierra Nevada will be the result of complex interactions between climate and weather, ecosystem and disturbance processes, and land management, all driven by accelerating climate change. Past experience provides inadequate analogues for interactions between climate-driven increases in disturbance magnitude and frequency. Our results demonstrate that by incorporating dynamic vegetation feedbacks to fire and carbon, we can better constrain our understanding of potential futures in the Sierra Nevada. However, the potential for extreme climatic events and their effect on disturbance processes such as widespread beetle and drought-related tree mortality, subsequent wildfire, and resulting forest succession pathways, assure us of surprises ahead.

## Methods

### Study Area

We conducted this study using three transects across the Sierra Nevada Mountains of California and Nevada. The three transects capture the elevation and latitudinal gradient present in the Sierra Nevada (Supplementary Fig. S1). The range of the elevation gradients decreases from the south (290–4388 m) to the central (252–3978 m) to the north (275–2591 m) in the Sierra Nevada. Tree species distributions vary as a function of latitude and elevation. Precipitation-limited low elevation woodlands and forests are dominated by oaks (*Quercus* spp.) and a mix of gray pine (*Pinus sabiniana*) and ponderosa pine (*P*. *ponderosa*)^24^. Mid-elevation forests, which typically occupy the area at or above the winter-persistent snowline, are primarily conifer-dominated and include a mix of white fir (*Abies concolor*), Douglas-fir (*Pseudotsuga menziesii*), ponderosa pine, Jeffrey pine (*P*. *jeffreyi*), sugar pine (*P*. *lambertiana*), and incense-cedar (*Calocedrus decurrens*)^24^. The upper montane and subalpine forests are comprised of mixes and pure stands of red fir (*A*. *magnifica*), western white pine (*P*. *monticola*), mountain hemlock (*Tsuga mertensiana*), lodgepole pine (*P*. *contorta*), and whitebark pine (*P*. *albicaulis*)^24^. Lower-elevation woodlands on the eastern side of the mountain range are primarily occupied by pinyon pine (*P*. *monophylla*)^24^.

### Model

We simulated forest dynamics under projected climate and wildfire using the LANDIS-II forest succession and disturbance model, with the Century Succession extension^25^ (v3.1.1) to simulate carbon pools and fluxes and the Dynamic Leaf Biomass Fuel extension^26^ (v2.0) and Dynamic Fire extension^26^ (v.2.0.5) to simulate wildfire and fuels. The model was previously parameterized and validated for our study area^16,21^. While the model does not account for increasing atmospheric CO_2_, empirical evidence indicates that CO_2_ fertilization effects are less likely as a result of N limitation^27^.

We used 12 km downscaled climate projections from the Intergovernmental Panel on Climate Change Fourth Assessment Report^28–30^. The models were forced with the A2 emission scenario. We used projections from CNRM CMS (Centre National de Recherches Météorologiques Coupled Global Climate Model), CCSM3 (National Center for Atmospheric Research Community Climate System Model), and GFDL CM2.1 (Geophysical Fluid Dynamics Lab coupled model). These climate models performed well in capturing both climate variability and seasonality over the historical period in California^31^.

The Dynamic Fire extension functions such that at each time-step, random ignitions occur on the landscape. When an ignition occurs in a grid cell, a draw from the fire probability distribution determines if the ignition becomes a fire. We used the same distribution as Liang *et al*.^16,18,21^, which was based on contemporary fire probabilities. If the ignition becomes a fire, then a draw from the fire size distribution determines the maximum size of the individual fire. The simulated fire size is influenced by a draw from the fire weather distribution and the biomass available to burn where the fire ignites. In prior studies^16,18,21^ and for our static simulations in this study, we used climate model-specific fire size projections at a 12 km resolution for large wildfires (>200 ha) developed by Westerling *et al*.^2^. Area burned is modeled using generalized Pareto distributions of log-area burned from fires occurring from (1984–2014), conditional on cumulative monthly moisture deficit from climate projections and biomass simulated for the historic period. For our dynamic simulations, which account for area burned in the prior decade, we updated biomass every ten years with simulated biomass from the dynamic vegetation model forced with simulated climate and fire. At each decadal time-step, we used the aboveground biomass layer from the prior decade’s simulation as an input to the fire size distribution model. This iterative approach resulted in decadal fire size distributions that were either a function of projected climate (static) or projected climate and prior area burned (dynamic). Prior fire events impact both the distribution of live and dead biomass and the fuel model associated with a burned grid cell. Non-forested grid cells are assigned a fuel model that has a rate-of-spread associated with grassland vegetation.

### Emissions

We estimated emissions using the FINN model algorithm^32^. Emissions of major gaseous and particulate species were calculated using dry biomass burned from wildfires and emission factors estimated for the western U.S. wildfires^33^. We derived dry biomass burned in kilograms at each time-step based on fire C efflux output from the Dynamic Fire extension. We used emission factors (g Kg^−1^) of carbon dioxide (CO_2_, 1454), carbon monoxide (CO, 89.3), methane (CH_4_, 4.9), submicron aerosols (PM1, 26), and organic aerosols (OA, 24.3) from Liu *et al*.^33^. The emissions factors are a mean from western US wildfires.

### Analysis

Fire size distributions by time period aggregated all simulated fires for the three transects. We used Welch two sample t-test on log-transformed fire sizes to compare the static and dynamic fire size distributions. To scale simulation results from our transects to the entire Sierra Nevada, we calculated area-weighted estimates by applying area-weighted mean values by vegetation type from the three transects to the entire mountain range (3.4 × 10^6^ ha). All simulation data were processed and analyzed using R^34^, including the raster^35^ package. We used the ggplot2^36^ to create figures.

## Supplementary information

Supplemental Material

## Data Availability

Simulation output data and code used in this study (10.25827/m7nr-ft88) are available at https://digitalrepository.unm.edu/bio_data/1/.
