# Supplementary material for: Vegetation-fire feedback reduces projected area burned under climate change
Source: Sci Rep. 2019 Feb 26;9:2838. doi: 10.1038/s41598-019-39284-1 (PMC6391438; doi:10.1038/s41598-019-39284-1)
Supplement: Supplementary file 1 — Supplemental Material [file 41598_2019_39284_MOESM1_ESM.docx]

**Supplementary Material**

Vegetation-fire feedback reduces projected area burned under climate change

Matthew D. Hurteau*, Shuang Liang, A. LeRoy Westerling, Christine Wiedinmyer

*corresponding author. mhurteau@unm.edu

Table S1: Summary statistics in hectares for static and dynamic fire size distributions for all simulated fires by time period. Within each time period fire sizes have been aggregated from three transects (North, Central, South), three climate models, and ten replicate simulations.

|  | Early (2010-2039) | | Mid (2040-2069) | | Late (2070-2099) | |
| --- | --- | --- | --- | --- | --- | --- |
|  | Dynamic (ha) | Static (ha) | Dynamic (ha) | Static (ha) | Dynamic (ha) | Static (ha) |
| Minimum | 2.25 | 2.25 | 2.25 | 2.25 | 2.25 | 2.25 |
| 1^st^ Quartile | 182.25 | 187.31 | 187.88 | 189.00 | 178.88 | 191.25 |
| Median | 461.25 | 474.75 | 438.75 | 474.75 | 443.25 | 515.25 |
| Mean | 1060.05 | 1105.49 | 1003.24 | 1181.99 | 1071.17 | 1355.77 |
| 3^rd^ Quartile | 1079.44 | 1100.25 | 1041.75 | 1163.25 | 1069.88 | 1316.25 |
| Maximum | 85167.00 | 50107.50 | 35253.00 | 41411.25 | 37482.75 | 178366.5 |

Table S2: Summary statistics in hectares for static and dynamic fire size distributions of the largest wildfires in each replicate simulation by time period. Within each time period fire sizes have been aggregated from three transects (North, Central, South), three climate models, and ten replicate simulations.

|  | Early (2010-2039) | | Mid (2040-2069) | | Late (2070-2099) | |
| --- | --- | --- | --- | --- | --- | --- |
|  | Dynamic (ha) | Static (ha) | Dynamic (ha) | Static (ha) | Dynamic (ha) | Static (ha) |
| Minimum | 5062 | 4028 | 3283 | 5402 | 6379 | 6311 |
| 1^st^ Quartile | 11448 | 10267 | 6888 | 11033 | 11288 | 11901 |
| Median | 14321 | 14270 | 9734 | 21606 | 15661 | 14892 |
| Mean | 23320 | 16618 | 12931 | 20257 | 16578 | 27011 |
| 3^rd^ Quartile | 27730 | 19689 | 15386 | 25118 | 20718 | 28931 |
| Maximum | 85167 | 50108 | 35253 | 41411 | 37483 | 178367 |


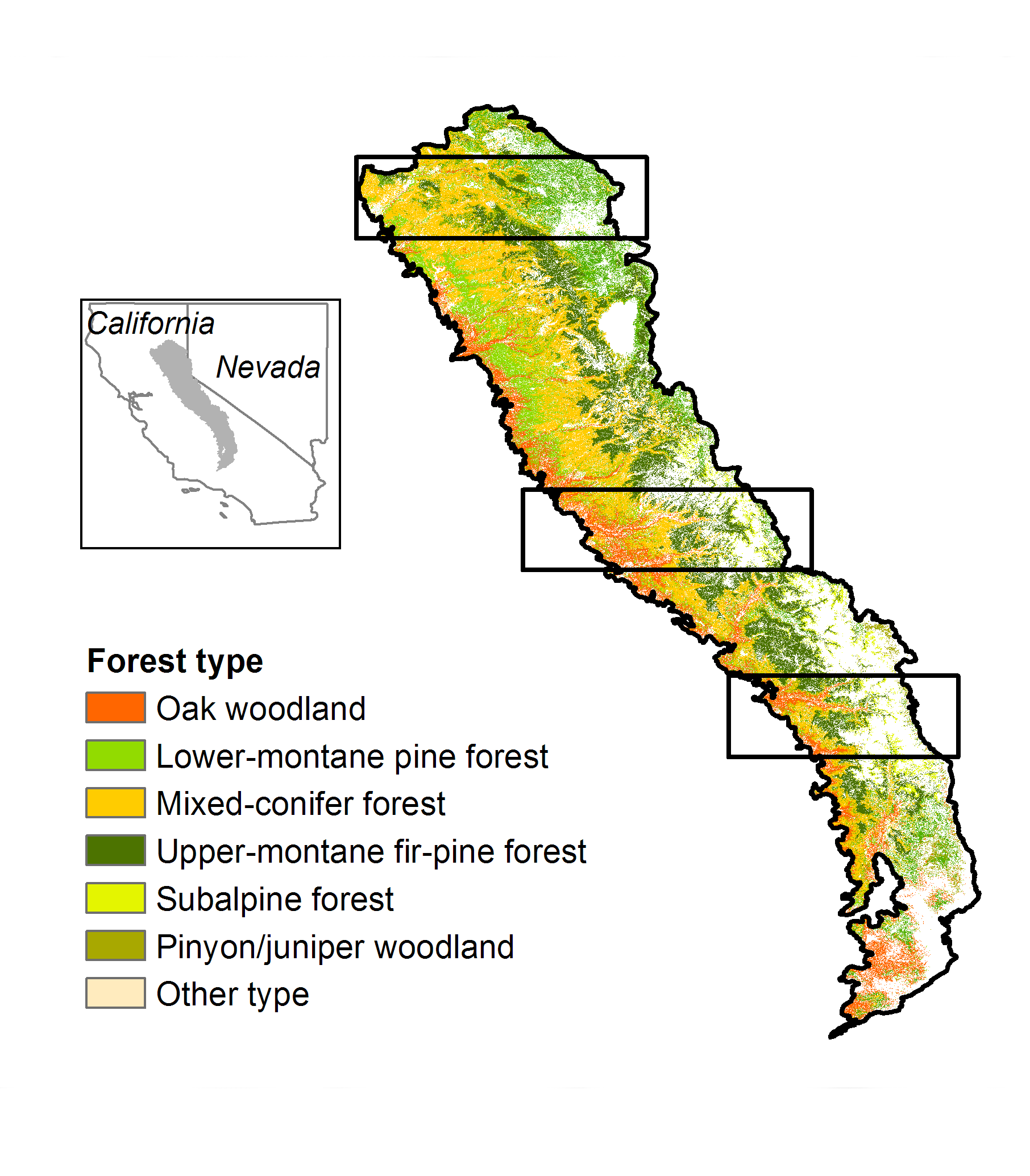


Fig. S1: Simulation domain of the three transects representing a latitudinal gradient along the Sierra Nevada Mountains. Colored background shows the initial distribution of simulated forest types derived from US Forest Service Forest Inventory and Analysis data.


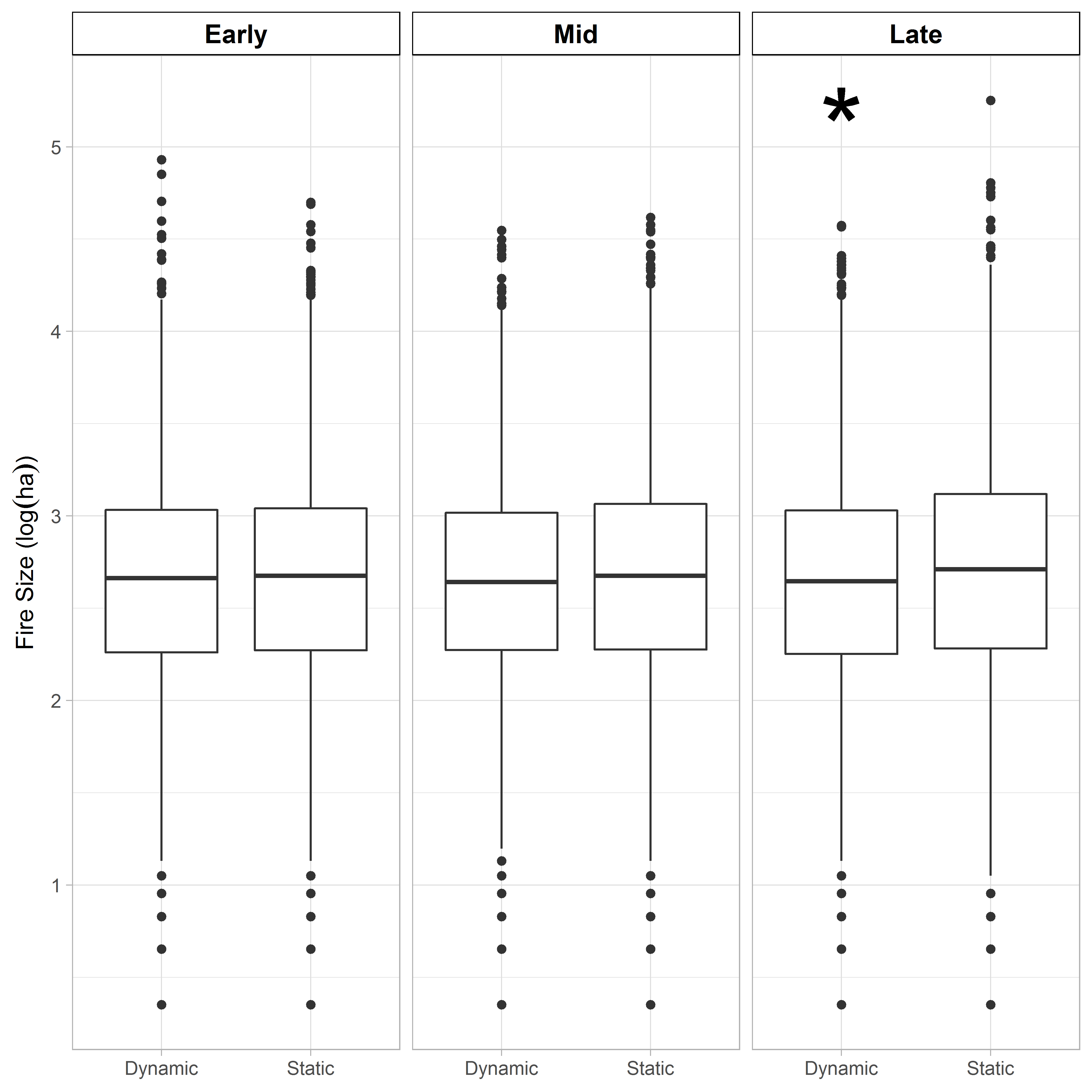


Fig. S2: Fire size distributions by 30-year period. The periods are 2010-39 (early), 2040-69 (mid), and 2070-99 (late). An asterisk denotes that the static distribution is significantly greater (p<0.001) than the dynamic distribution for that time period. The dynamic simulations include decadal re-estimated area burned distributions that account for prior fire events and projected climate. The static simulations include area burned distributions estimated only on projected climate.


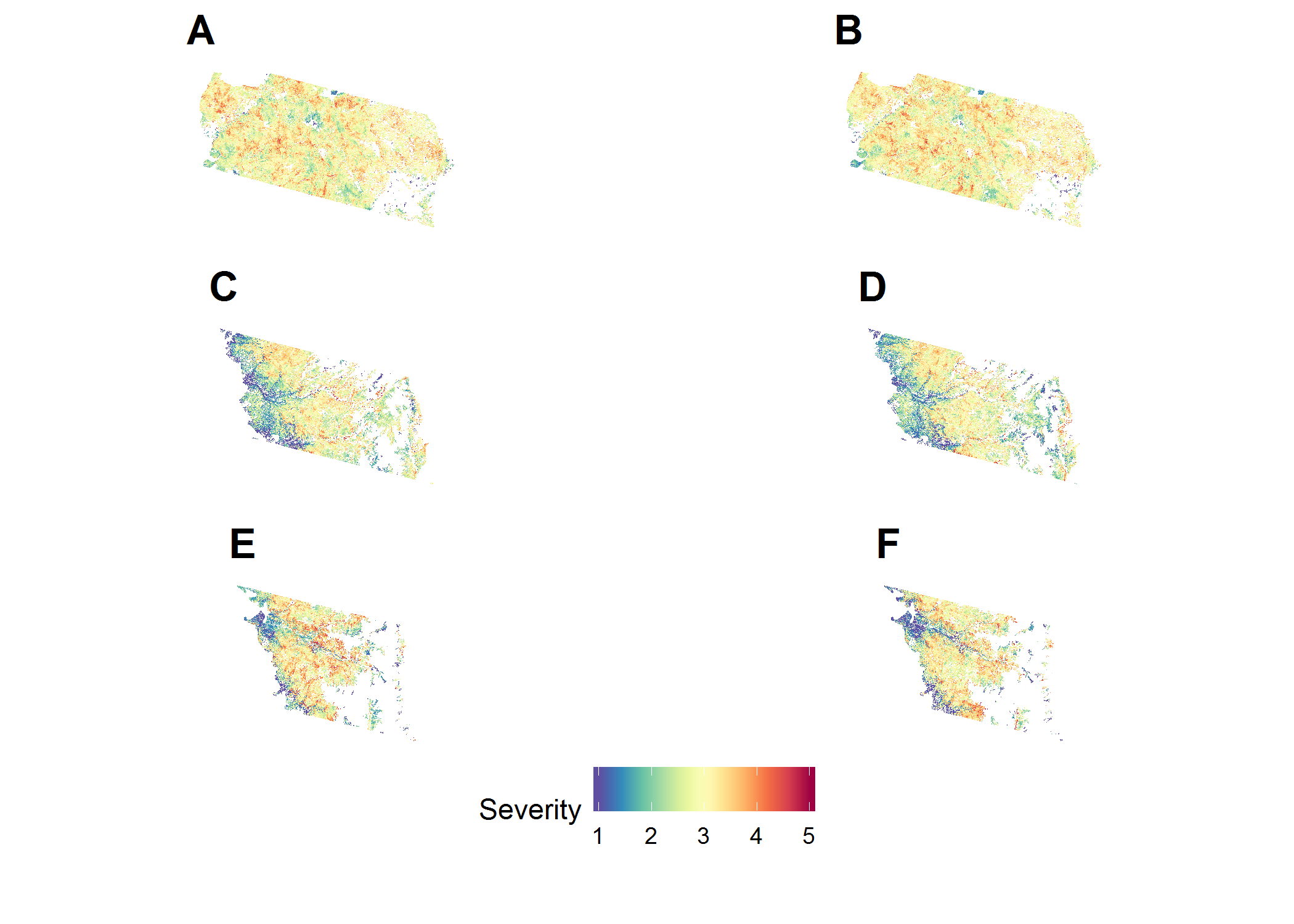


Fig. S3: Mean fire severity for the three transects across the Sierra Nevada Mountains (north: A, B; central: C, D; south: E, F) for static (A, C, E) and dynamic (B, D, F) fire simulations. The mean layers were calculated using all fire severity values in a given grid cell from all years of all replicate simulations. This includes three climate projections and ten replicates of each. Fire severity is scaled as surface fire (1-2), mixed severity fire with some torching (3), mixed severity fire with some crowning (4), and crown fire (5).


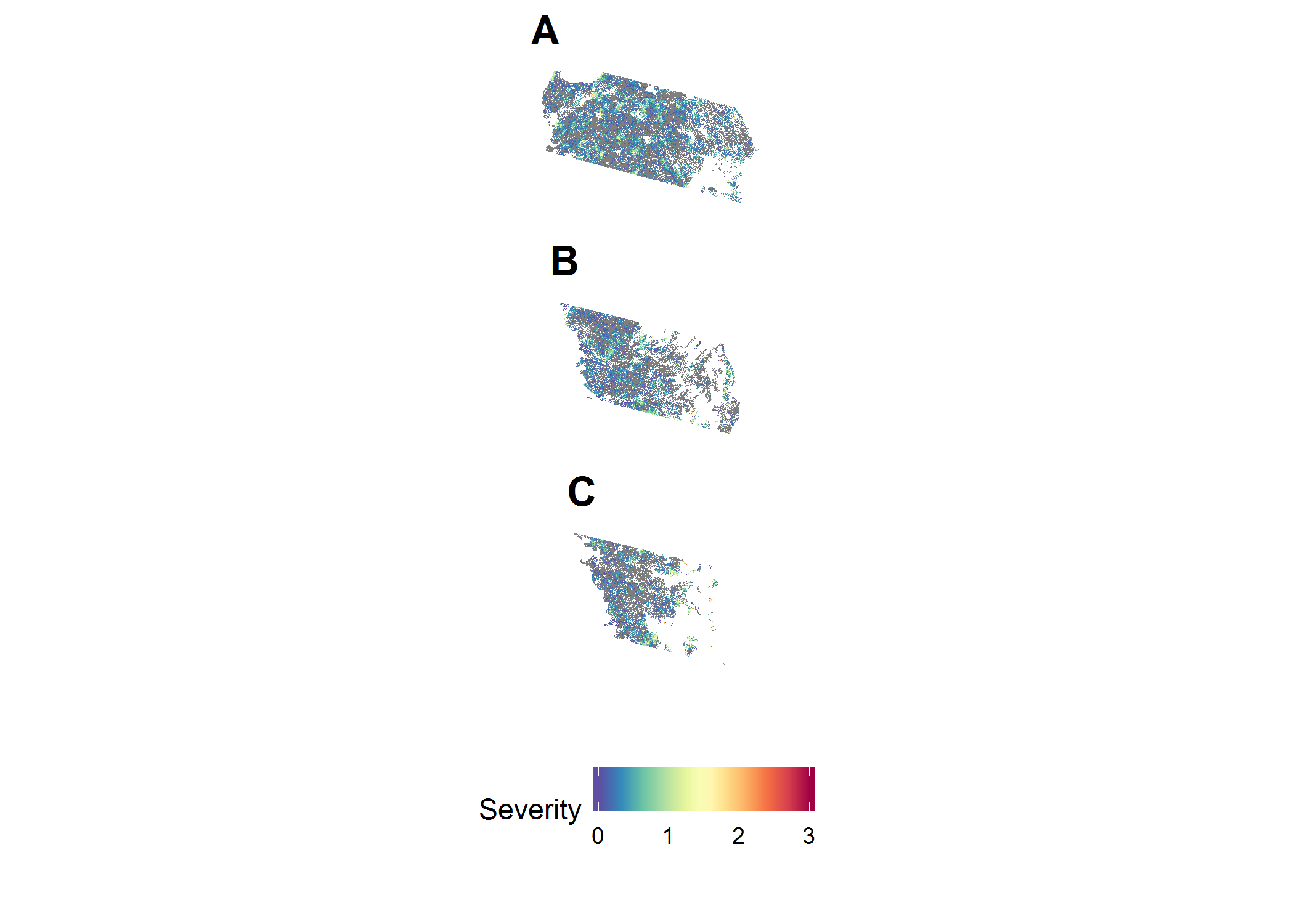


Fig. S4: The difference in mean fire severity between the static and dynamic fire simulations for the three transects across the Sierra Nevada Mountains (north: A; central: B; south: C). Differences were calculated by subtracting the mean dynamic layer from the mean static layer presented in supplemental figure S3.


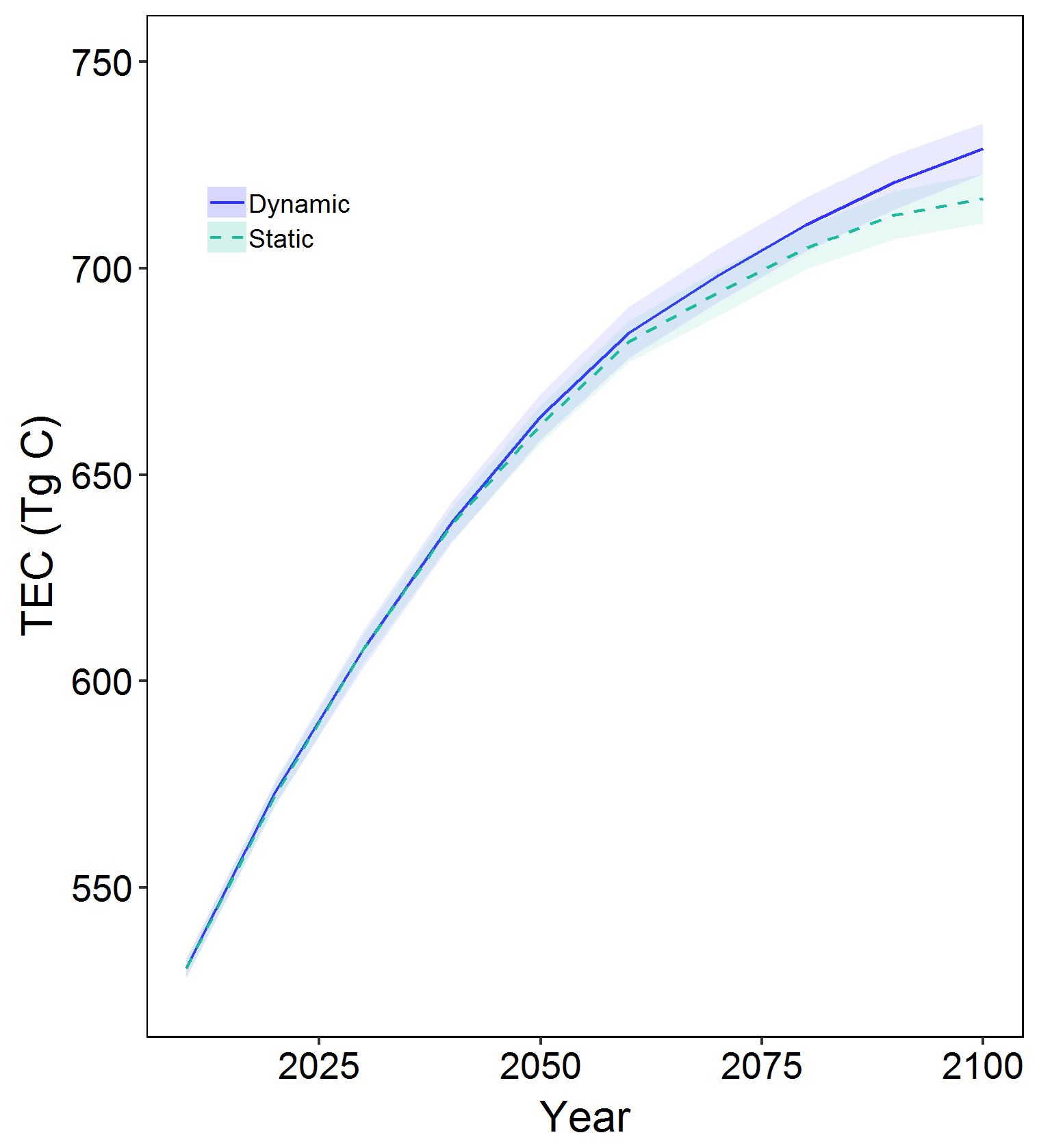


Fig. S5: Total ecosystem carbon (TEC) for the Sierra Nevada. The dynamic simulations include decadal re-estimated area burned distributions that account for prior fire events and projected climate. The static simulations include area burned distributions estimated only on projected climate. Lines are means and shaded area the 95% confidence intervals for area-weighted values calculated from simulation results on three transects, representing a latitudinal gradient across the Sierra Nevada.
